# The impact of anxiety on chronic musculoskeletal pain and the role of astrocyte activation

**DOI:** 10.1097/j.pain.0000000000001445

**Published:** 2018-12-05

**Authors:** James J. Burston, Ana M. Valdes, Stephen G. Woodhams, Paul I. Mapp, Joanne Stocks, David J.G. Watson, Peter R.W. Gowler, Luting Xu, Devi R. Sagar, Gwen Fernandes, Nadia Frowd, Laura Marshall, Weiya Zhang, Michael Doherty, David A. Walsh, Victoria Chapman

**Affiliations:** a Arthritis Research UK Pain Centre, University of Nottingham, Medical School, Queen's Medical Centre, Nottingham, United Kingdom; b School of Life Sciences, University of Nottingham, Nottingham, United Kingdom; c School of Medicine, University of Nottingham, Nottingham, United Kingdom; d NIHR Nottingham Biomedical Research Centre, University of Nottingham, Nottingham, United Kingdom; e Arthritis Research UK Centre for Sport, Exercise and Osteoarthritis, University of Nottingham, Nottingham, United Kingdom

## Abstract

Anxiety and depression are associated with increased pain responses in chronic pain states. The extent to which anxiety drives chronic pain, or vice versa, remains an important question that has implications for analgesic treatment strategies. Here, the effect of existing anxiety on future osteoarthritis (OA) pain was investigated, and potential mechanisms were studied in an animal model. Pressure pain detection thresholds, anxiety, and depression were assessed in people with (n = 130) or without (n = 100) painful knee OA. Separately, knee pain and anxiety scores were also measured twice over 12 months in 4730 individuals recruited from the general population. A preclinical investigation of a model of OA pain in normo-anxiety Sprague-Dawley (SD) and high-anxiety Wistar Kyoto (WKY) rats assessed underlying neurobiological mechanisms. Higher anxiety, independently from depression, was associated with significantly lower pressure pain detection thresholds at sites local to (*P* < 0.01) and distant from (*P* < 0.05) the painful knee in patients with OA. Separately, high anxiety scores predicted increased risk of knee pain onset in 3274 originally pain-free people over the 1-year period (odds ratio = 1.71; 95% confidence interval = 1.25-2.34, *P* < 0.00083). Similarly, WKY rats developed significantly lower ipsilateral and contralateral hind paw withdrawal thresholds in the monosodium iodoacetate model of OA pain, compared with SD rats (*P* = 0.0005). Linear regressions revealed that baseline anxiety-like behaviour was predictive of lowered paw withdrawal thresholds in WKY rats, mirroring the human data. This augmented pain phenotype was significantly associated with increased glial fibrillary acidic protein immunofluorescence in pain-associated brain regions, identifying supraspinal astrocyte activation as a significant mechanism underlying anxiety-augmented pain behaviour.

## Abstract 2

Supplemental Digital Content is Available in the Text.

Anxiety predicts onset of knee pain and drives greater osteoarthritis pain in humans. Our validated preclinical model identifies supraspinal astrocytosis as a potential mechanism.

## 1. Introduction

Chronic pain remains a global clinical problem. Negative affect, including anxiety and depression, is associated with lower pain thresholds in healthy individuals^52^ and is exacerbated in chronic pain states,^13^ including musculoskeletal disorders such as low back pain.^7,53^ Negative affect is also common in people with osteoarthritis (OA),^3^ the most prevalent joint disease and a major cause of disability and chronic musculoskeletal pain.^14^ Both anxiety and depression have direct effects on reported current OA pain, and pain reported the following week.^45^ Whether anxiety predicts long-term worsening of OA pain, facilitation of pain, and/or a spread of pain to remote nondiseased areas^2,37,59^ remains unknown. The association of negative affect with greater use of opioid analgesics^5,38,54^ and worse treatment outcomes to arthroplasty^1,35,56^ warrants further investigation of the impact of negative effect on OA pain.

Multiple brain regions have fundamental roles in the processing of negative affect and chronic pain. Among these, the periaqueductal gray (PAG) and anterior cingulate cortex (ACC) have well-documented roles in anxiety,^12,31^ and both regions are activated in people with OA pain.^4,19^ Human imaging studies revealed ACC activation during anticipation of pain, which was correlated with the level of anxiety–pain interactions.^58^ The PAG, a major component of a descending pain system, normally provides tonic inhibition of spinal cord nociceptive responses.^29^ Imaging studies in OA subjects revealed differences in activity in brainstem regions including the PAG, which were related to pain pressure thresholds,^19^ and neural correlates of reported OA pain intensity mapped to limbic affective circuits were explained by trait anxiety.^11^

We hypothesised that higher anxiety scores are associated with high OA pain scores and spread of pain to nondiseased areas. To test this, quantitative sensory testing (QST) was used to measure pressure pain detection thresholds (PPTs), alongside anxiety and depression scores in people with or without painful knee OA. Whether preexisting anxiety contributed to future knee pain severity was also evaluated using questionnaire data from a community cohort at 2 time points.

The disconnect between the preclinical efficacy of candidate treatments and success in the clinic is frequently attributed to a preclinical focus on sensory pain, which fails to account for the contribution of affective responses.^9^ Thus, the second part of this study back-translated our clinical findings to investigate the underlying neurobiological mechanisms in a rodent model of negative affect. Wistar Kyoto (WKY) rats exhibit heightened anxiety/depression, increased stress-induced behavioural responses,^8^ and hypothalamic–pituitary–adrenal axis activation.^46^ Rat models of OA pain mimic key histopathological features of human OA,^55^ and exhibit spinal neuronal hyperexcitability^43^ and glial cell activation.^42,60^ Using the WKY strain of rats to model comorbid negative affect and OA pain, we investigated the potential contribution of astrocytes (using glial fibrillary acidic protein [GFAP] immunofluorescence) at spinal and supraspinal sites to altered OA pain responses. Our novel findings have important implications for the identification of novel targets for treatments and the use of antianxiety medications as analgesic adjuvants in OA pain.

## 2. Methods

### 2.1. Clinical subjects

Participants from a community-based cohort study aged 40 years and older (“Knee Pain and Related Health in the Community” study)^16^ were recruited by post through general practice registers in the East Midlands. This study was approved by the Nottingham Research Ethics Committee 1 (NREC Ref: 14/EM/0015) and registered (clinicaltrials.gov portal: NCT02098070) in line with the Declaration of Helsinki. Participants provided written informed consent and completed a questionnaire evaluation including the Hospital Anxiety and Depression Scale (HADS), which has been validated against a structured clinical interview by a liaison psychiatrist of depression or anxiety in people with OA,^3^ and a knee pain questionnaire. A subset of participants were invited to undergo a hospital-based assessment involving knee radiographs and PPTs on both knees and the sternum.

Associations between knee pain, anxiety, and depression scores were tested using questionnaire data from 4730 community-recruited individuals, of whom 3274 participants without knee pain at baseline also underwent a 12-month follow-up. A subgroup of 230 participants were selected for PPT assessments—130 individuals with current knee pain (present most days, lasting more than 3 months) and radiographic knee OA (Kellgren–Lawrence [KL] radiographic score >2 in the tibiofemoral [TF] or patellofemoral [PF] compartments of either knee), plus 100 knee pain–free individuals without radiographic knee OA.

### 2.2. Pressure pain detection thresholds

Quantitative sensory testing for PPTs was performed on individuals using an electronic pressure algometer connected to a laptop and a patient switch (Somedic Sensebox; Somedic SenseLabs, Sösdala, Sweden). Quantitative sensory testing was conducted at the medial TF joint line of the most painful knee, or the right knee if there was no pain in either, and also at sites distal to (anterior tibia) and distant from (sternum) the index knee. Pressure pain detection threshold assessment has been standardized by ourselves, and others, at these sites in people with OA^47,51^.

Participants were first familiarized with the procedure by applying the stimulus to a learning site on one hand. Pressure pain detection thresholds were then measured sequentially at sternum, medial knee, and anterior tibia. For each test, the pressure (kPa) was recorded at which pain was first experienced during application of progressively increasing pressure using a probe with a 1 cm^2^ blunt end and a constant ramp of 50 kPa per second.^37^ The test stimulus was applied to each site 3 times.

The intraclass correlation coefficient for 2 raters was calculated taking the log of the pressure pain thresholds measured in 9 independent subjects. The intraclass correlation coefficients (95% confidence intervals [CIs]) obtained were as follows: anterior tibia 0.69 (0.32-0.87), knee lateral 0.85 (0.63-0.94), knee medial 0.81 (0.56-92), and sternum 0.64 (0.26-0.85). The medial, anterior tibia, and lateral PPTs refer to the index knee, which was a knee at random for controls, the only painful knee for individuals with unilateral knee pain, and the most painful knee for individuals with bilateral knee OA, respectively.

### 2.3. Radiographic evaluation

Tibiofemoral and PF radiographs were taken using a standardised protocol [standing posterior–anterior and skyline views] and scored by experienced observers with intrarater and interrater reliability tests performed. A Perspex Rosenberg template with lead beads was used for the standing posterior–anterior view to standardise the degree of knee flexion, foot rotation, and magnification.^41^ Posterior–anterior radiographs were taken with the patient facing the x-ray tube while standing on the Rosenberg jig and leaning forwards with their thighs touching the anterior aspect of the jig, the x-ray beams passing from the posterior aspect through to the anterior aspect of the knee. Variable jigs were used for the skyline view to obtain 30° of knee flexion with the participant lying in a reclined supine position on a couch. Grading of radiographs for changes of OA included the summated KL score and the Nottingham logically devised line drawing atlas for individual scoring of osteophyte (0-5) and joint space width (−1 to +5) for each medial TF, lateral TF, and PF compartment. Individuals were classified as OA positive if they had a KL grade ≥2 at either compartment in one or both knees, based on the above definition.

### 2.4. Questionnaire evaluation

The HADS is a self-screening, 14-item questionnaire incorporating anxiety and depression subscales, which is extensively used in primary care.^3^ The anxiety and depression subscales can each take values ranging from 0 to 21 and scores are categorised as normal (0-7), mild (8-10), moderate (11-14), and severe (15-21). For the current study, we used the standard cutoff of ≤10 between normal/mild for low anxiety or depression and >10 for moderate or severe.^62^

The Intermittent and Constant Osteoarthritis Pain (ICOAP) scale is an 11-item questionnaire, divided into 2 domains: a first 5-item scale for constant pain and a 6-item scale for intermittent pain (so-called “pain that comes and goes”). Each domain captures pain intensity as well as related distress and the impact of OA pain on quality of life. Preliminary data suggested the new measure to be valid and reliable.^20^ For the current analysis, we only used the pain intensity of the constant and intermittent pain domains. A 0 to 10 numerical rating scale (NRS) was used for self-assessment of pain intensity in the past month.

### 2.5. Statistical analysis

Questionnaire data outcomes were: HADS anxiety score >10 at 12 months in individuals with baseline anxiety scores <9 and presence of knee pain >15 days of the month and lasting at least 3 months in individuals with no knee pain at baseline. Pressure pain detection threshold values for sternum, knee medial, knee lateral, and anterior tibia are expressed in log10 kPa. Additional outcomes were pain intensity measures for constant and intermittent pain (0-5) and a 0 to 10 NRS to assess pain intensity in the past month. Associations between pain outcomes and HADS anxiety and depression status were tested using standard linear regressions. Correlations between anxiety and depression HADS scores and other quantitative variables were determined using Spearman correlation coefficient. Age, sex, and body mass index were included as covariates in all analyses. Other covariates included are specifically mentioned in the text. Association is expressed as the linear regression coefficient beta and the corresponding 95% CIs; *P* < 0.05 was considered statistically significant. Analyses were performed in R (version 3.1.2; the R Foundation for Statistical Computing, Vienna, Austria). The study was powered to detect with 80% power, and *P* < 0.05 differences of 0.57 StDev in knee OA and of 0.93 StDev in controls between high vs low anxiety scores. These values correspond to 0.16 and 0.21 log kPa in knee OA and controls, respectively. Because the effect size for PPTs in healthy controls is about half that seen in OA cases, this impacts on the power to detect a statistically significant (<40%) difference in the control group.

### 2.6. Preclinical model of osteoarthritis pain and anxiety

Studies were in accordance with UK Home Office Animals (Scientific Procedures) Act (1986) and ARRIVE guidelines.^27^ Ninety-one male rats were used: Sprague-Dawley (SD) n = 16, Wistar n = 17 (Charles River, Margate, United Kingdom), Wistar Kyoto (WKY) n = 57 (Envigo, Bicester, United Kingdom). Rats were housed in groups of 3 to 4 on a 12-hour light–dark cycle in a specific pathogen-free environment with ad libitum access to standard rat chow and water. Rats were randomly assigned to groups, and experimenters were blinded to all treatments. Males were used to reduce variability, and thus the number of animals required, and maintain consistency with previous work characterising the monosodium iodoacetate (MIA) model in SD rats. When comparing more detailed longitudinal changes in anxiety-like behaviour in WKY rats, Wistar rats were used as the most genetically similar control strain.

### 2.7. Osteoarthritis model and behavioural assessments

Unilateral intra-articular injection of (MIA) was used to model OA pain in adult male SD and WKY rats (MIA n = 10; saline n = 6 per strain). Rats were anesthetised (isoflurane 2.5%-3% in 1 L/minutes O_2_) and received a single intra-articular injection of 1 mg of MIA (Sigma, United Kingdom) in 50 μL saline, or saline alone, through the infrapatellar ligament of the left knee.^43^ Group allocations were randomised by an independent investigator. The MIA model was chosen over surgical models to minimise the impact of surgical pain not related to OA in this study. The MIA model mimics key elements of joint pathology and clinical OA pain (see references in Ref. 6).

Weight-bearing asymmetry was measured with an incapacitance tester (Linton Instrumentation, Diss, United Kingdom). Paw withdrawal thresholds (PWTs) were determined for each hind paw using the up-down method as previously described.^42^ Briefly, von Frey hairs (vFH; Semmes-Weinstein; bending forces 0.4-26 g) were applied to the plantar surface of the hind paw for 3 seconds and the presence or absence of a withdrawal response was recorded. Pain behaviour was assessed at baseline, and then 2 to 3 times per week until 28 days after MIA or saline injection. Joint pathology was assessed at the end of experiments, as previously described.^33^ Tibiofemoral joints were removed, postfixed in neutral buffered formalin (10%), decalcified in EDTA, then processed, and stained with haematoxylin and eosin, to enable scoring of joint pathology. Cartilage surface integrity was scored from 0 (healthy) to 5 (full-thickness degeneration), and a total joint damage score (range 0-15) was calculated as cartilage surface integrity score (0-5)  ×  proportion of damaged cartilage (0-3). Inflammation was graded on a scale from 0 (lining cell layer 1-2 cells thick) to 3 (lining cell layer >9 cells thick and/or severe increase in cellularity).

Anxiety-like behaviour was determined via time in the central zone of an open-field arena for comparisons between SD and WKY rats. Sprague-Dawley and WKY rats were placed in a corner of an open-field testing arena, comprising an opaque white plastic box (60 cm × 63 cm, wall height 20 cm). Behaviour was then assessed for a period of 5 minutes, with total duration in the central zone (20 cm × 20 cm), frequency of entries to the central zone, and total distance moved quantified using EthoVision software (Noldus Information Technology; the Netherlands^25^) or manual scoring. More detailed examination of the association between pain and anxiety-like behaviour in Wistar and WKY rats was assessed via area under the curve (AUC) for time spent in the open arm in the elevated plus maze (EPM) at baseline and 18 to 21 days after intra-articular injection of saline (Wistar: n = 6, WKY: n = 8) or MIA (Wistar: n = 11, WKY: n = 7). To control for any strain differences in locomotor activity, total distance travelled was assessed in the EPM and via the number of beam breaks in locomotor activity boxes. To prevent habituation to the testing equipment, the maze was rotated 90° and visual cues in the room altered for the second measurement. Anxiety status was determined by comparing individual AUC values to the mean AUC value for all animals at each time point. Individual animals with AUC values below the mean value were considered anxious and those with values above the mean value were considered nonanxious. These data were used for linear regression analyses.

### 2.8. Immunohistochemical analysis of glial cell activation

Perfusion-fixed spinal cord and brain tissue were collected and glial cell activation was analysed as previously described^42^ in lumbar dorsal horn of the spinal cord, PAG, and ACC.

Briefly, 40-µm spinal cord sections were immunolabelled with primary antibodies against ionized calcium binding adapter molecule (IBA1; rabbit, 1:1000, Wako, Neuss, Germany) for microglia, and GFAP (mouse, 1:100; Fisher Scientific, Loughborough, United Kingdom) for astrocytes. Activated microglia with pronounced swelling of the soma and reduced ramified process number were considered activated, and the total number of these cells expressing IBA1 (goat 1:100; Abcam, Cambridge, United Kingdom, ab5076) and phosphorylated-p38 (P-p38; 1:300, Cell Signalling, 9211) and P-p38 were counted (n = 5 sections/animal, n = 5 animals/group). In brain, 20-µm sagittal cryosections containing PAG and ACC were immunolabelled for GFAP (rabbit, 1:100; DAKO, Cambridge, United Kingdom). Primary antibodies were detected with Alexa Fluor 488 goat anti-rabbit (IBA-1, P-p38) or Alexa Fluor 568 goat anti-mouse (GFAP), all at 1:300 (Molecular Probes, Eugene, OR), and sections were imaged using a 20 × 0.4 NA objective lens on a Leica DMIRE2 fluorescence microscope. For GFAP figures, additional representative high-quality images of GFAP labelling for each brain region were obtained using a Zeiss LSM880C confocal microscope (20 × 0.5 NA objective). Briefly, a z-stack of 12 images collected at 0.5-µm intervals was obtained from one section per animal, with identical acquisition settings, and maximum-intensity projections were generated using ImageJ.

### 2.9. Quantification of glial cell activation

Microglia were morphologically assessed for activation status by an experimenter blinded to the treatment. IBA-1–positive cells were considered activated if they displayed pronounced swelling of the cell body and significantly reduced ramified process number. The total number of contralateral dorsal horn–activated microglia was subtracted from the ipsilateral total to give a measure of MIA-induced change in microglial activation. Similarly, for P-p38, the total number of DAPI-positive cells expressing P-p38 was manually counted.

For GFAP immunofluorescence, number of pixels with mean gray intensity >45 was determined for each region using Volocity 5.5 software.

### 2.10. Duloxetine intervention

To probe mechanisms linking OA pain behaviour, negative affect, and brain glial cell responses, effects of the serotonin and noradrenaline reuptake inhibitor (SNRI) duloxetine were studied. Duloxetine is an effective analgesic in 30% to 50% of patients with OA knee pain,^57^ a similar proportion to the 40% demonstrating symptoms of negative affect.^3^ These data suggest that treatments targeting the interaction between negative affect and pain may have greater utility in this subpopulation of OA sufferers. WKY rats were injected with MIA (n = 20) and randomly stratified into drug or vehicle treatment at day 14. At day 20, duloxetine (30 mg/kg, subcutaneous, n = 10) or vehicle (1% ethanol, 1% Tween 80 in saline, n = 10) was injected once daily for 3 days, and pain behaviour was compared to WKY/saline rats treated with vehicle (n = 6). On day 22, pain behaviour was assessed 2.5 hours after treatment, tissue collected, and GFAP expression analysed. The dose of duloxetine was based on previous evidence of analgesic efficacy in rodent models of pain.^23,50^

### 2.11. Statistical analyses

Data were analysed using Prism 5.0 software (GraphPad, La Jolla, CA) with 1-way or 2-way analysis of variance tests with Bonferroni post hoc testing, or unpaired two-tailed *t* tests, as appropriate, and are reported as mean ± SEM (parametric data), or median ± interquartile range (nonparametric data). *P* < 0.05 was considered statistically significant. Associations between pain and anxiety scores were assessed via standard linear regression models using binary thresholds for pain and anxiety. Ipsilateral pain was defined as a change of ≥−3 vFH PWT from baseline; contralateral pain as ≥−2 vFH difference; and anxiety as open arm AUC < mean of all animals. Immunofluorescence data were analysed using nonparametric Kruskal–Wallis tests with Dunn post hoc testing. Correlations between GFAP intensity and pain behaviour were determined using Spearman correlation.

## 3. Results

### 3.1. Clinical data

#### 3.1.1. Anxiety and depression: higher pain scores and lower pain pressure thresholds in people with knee OA

The clinical and demographic characteristics of study participants are presented in Table 1. Pressure pain detection thresholds at each of the 4 sites were lower in the OA cohort than in the pain-free cohort (Table 1). Hospital Anxiety and Depression Scale anxiety scores were correlated with depression scores in the whole sample (Spearman's *R* = 0.58, *P* < 0.0001). Anxiety and depression scores were significantly higher in the OA cohort, with moderate or high anxiety reported in 25%, and depression in 10%, of OA cases (HADS scores >10) (Table 1). Scores were strongly correlated with self-reported pain and PPTs before any adjustments (data not shown), and remained significantly correlated after adjustment for covariates (Table S1, available at http://links.lww.com/PAIN/A696).

**Table 1:**
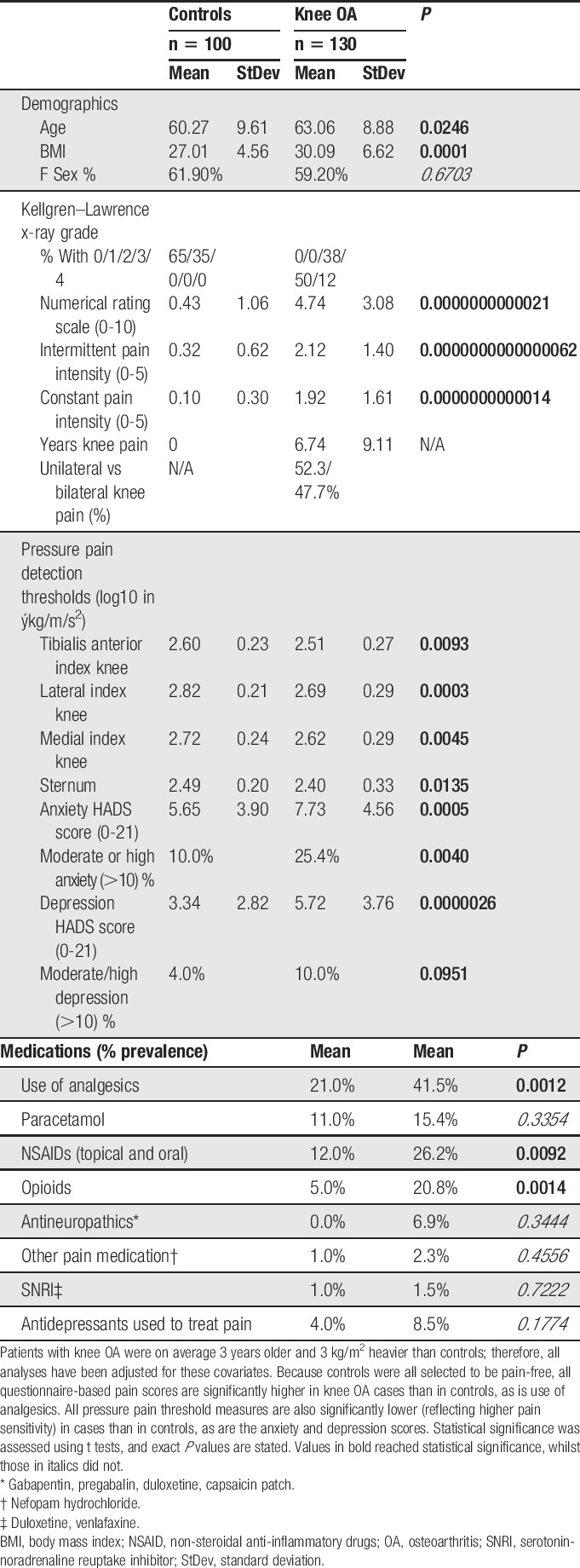
Demographic and clinical characteristics of study participants.

**Figure 1.**
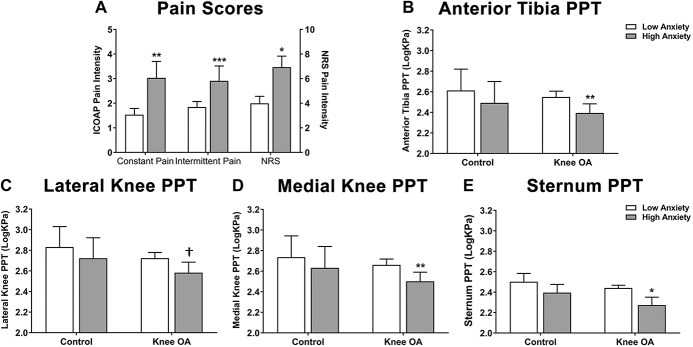
Association between anxiety status (low = HADS score ≤10; high = HADS score >10) and pain measures in patients with knee OA (n = 130) vs healthy controls (n = 100). Association between anxiety status and self-reported pain (A) in patients with knee OA, and pressure pain detection thresholds (PPTs) at the anterior tibia (B), and the lateral (C) and medial (D) aspects of the index knee, and at the sternum (E) in knee OA vs healthy controls. Data are mean log10 PPT ± SEM. *P*-values are derived from linear regression where pain scores or PPTs are the outcome and anxiety status (low or high) is the predictor variable, adjusting for age, sex, BMI, and depression status as per the analysis in Table 1. †*P* < 0.10, *P < 0.05, **P < 0.01, ***P < 0.0001. BMI, body mass index; HADS, Hospital Anxiety and Depression Scale; ICOAP, Intermittent and Constant Osteoarthritis Pain scale; OA, osteoarthritis.

Hospital Anxiety and Depression Scale anxiety and depression scores are not normally distributed; so, individuals were stratified into severe/moderate vs mild/normal anxiety and depression categories using previously validated cutoffs^3,21^ analysing cases and controls separately, and adjusting for covariates. The magnitude of pain scores for these groups is shown graphically in Figure 1. In individuals with painful knee OA, anxiety status was significantly associated with all 3 of the self-reported pain measures (Table 2 and Fig. 1A) and with PPTs at the medial and lateral knee, anterior tibia, and sternum (Table 2 and Figs. 1B–E). The coefficients for the association between anxiety and PPTs, and anxiety and self-reported pain in the knee OA cohort remained significant after adjustment for depression (Table 2). However, after adjustment for anxiety status, depression only remained associated with NRS pain intensity (Table 2). Neither anxiety nor depression status was significantly associated with PPTs in the pain-free cohort (Table 2 and Fig. 1).

**Table 2:**
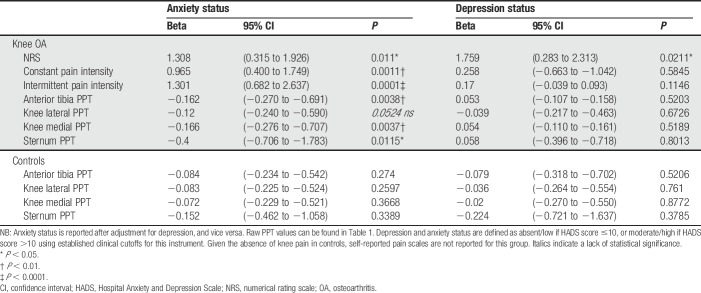
Association between pressure pain thresholds (PPTs), self-reported pain scores, anxiety, and depression status in knee OA cases and controls.

To investigate the potential temporal link between pain and anxiety, we used questionnaire data at 2 time points from 4730 individuals from the community. The effect of a HADS anxiety score >10 on the incidence of knee pain after 12 months was studied. Data from 3274 individuals who reported that they did not have knee pain at baseline (of whom 351 had a HADS anxiety score >10) were entered into a logistic regression model with the outcome being pain at 12 months (defined as presence of knee pain >15 days of the month and lasting at least 3 months). This analysis yielded an odds ratio (OR) of 1.96 (95% CI 1.46-2.61; *P* < 0.0000054). We further adjusted for the presence of depression as measured by HADS. This resulted in a slightly lower OR = 1.71 (95% CI 1.25-2.34 *P* < 0.00083). The effect of depression on knee pain onset, adjusted for anxiety, was OR = 1.66 (95% CI 1.09-2.53; *P* < 0.018).

A similar analysis was performed to investigate whether knee pain predicts the onset of anxiety 12 months later. Data from 3767 individuals with a baseline HADS anxiety score >10 were analysed, of which 1020 reported baseline knee pain. A logistic regression model including age, sex, and body mass index as covariates used baseline knee pain (15 days or more for the past month) as the predictor variable. This yielded an OR = 1.91 (95% CI 1.35-2.70 *P* < 0.010), which lowered to OR = 1.18 (95% CI 0.79-1.77 *P* < 0.40) when adjusted for baseline depression status. Assessing the effect of depression at baseline on the onset of anxiety (HADS > 10) 12 months later yielded OR = 3.20 (95% CI 1.69-6.09; *P* < 0.0004). These data demonstrate that both anxiety and depression at baseline are associated with increased risk of knee pain at follow-up. By contrast, knee pain at baseline was not significantly associated with onset of anxiety 12 months later.

### 3.2. Preclinical data

#### 3.2.1. Altered pain phenotype in a rodent model of high anxiety

**Figure 2.**
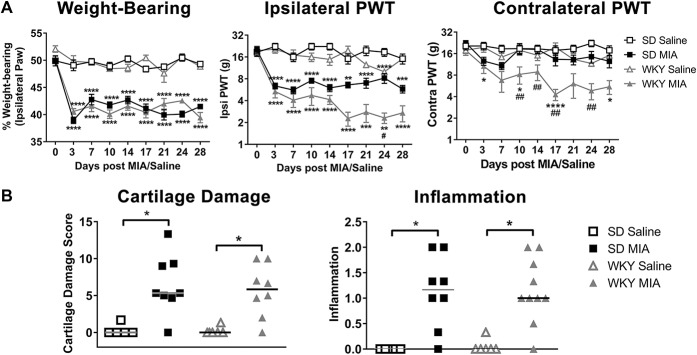
Augmented MIA-induced pain behaviour, but not knee pathology, in the WKY rat. (A) Intra-articular injection of MIA produced similar alterations in weight-bearing asymmetry in SD and WKY rats, compared with their respective saline-treated controls. However, a significantly greater decrease in ipsilateral and contralateral hind paw withdrawal thresholds (PWTs) was observed in WKY-MIA rats, demonstrating augmented pain behaviour in this strain. Data are mean ± SEM, 2-way analysis of variance with Bonferroni post hoc testing. *P < 0.05, **P < 0.01 ***P < 0.001 vs saline; #*P* < 0.05, ##*P* < 0.01 vs SD-MIA. (B) Enhanced MIA-induced pain behaviour in the WKY strain was not accompanied by altered joint pathology, with similar cartilage damage and joint inflammation scores compared to SD-MIA rats (n = 8). Data are medians, Kruskal–Wallis test with Dunn post hoc testing *P < 0.05. MIA, monosodium iodoacetate; SD, Sprague-Dawley.

To better understand the link between anxiety and knee pain, we established a model of OA pain in a rat strain with elevated baseline anxiety-like behaviour. Before model induction, WKY rats display increased anxiety behaviour in the open-field test (Figure S1, available at http://links.lww.com/PAIN/A696), but no significant differences in PWTs compared with normo-anxiety SD rats (Table S2, available at http://links.lww.com/PAIN/A696). After intra-articular injection of MIA to model OA pain, weight-bearing asymmetry (a measure of pain on loading) increased in both strains of rats. However, WKY rats showed significantly greater MIA-induced reductions in ipsilateral PWTs than SD rats, and also displayed a contralateral phenotype not observed in the SD strain, with significantly reduced contralateral PWTs from day 10 (Fig. 2A). Lowered PWTs are generally associated with the presence of spinal sensitization mechanisms.^32^ Monosodium iodoacetate–induced joint pathology was comparable between WKY and SD rats at day 28 (Fig. 2B), supporting a role of central mechanisms in mediating differences in pain responses.

Consistent with our clinical data, baseline scores of anxiety-like behaviour in the EPM predicted the MIA-induced change in contralateral PWTs (increased pain behaviour) in WKY rats (Table 3), mimicking key aspects of our clinical data and supporting the use of the WKY-MIA model to study the mechanisms by which anxiety may influence OA pain.

**Table 3:**
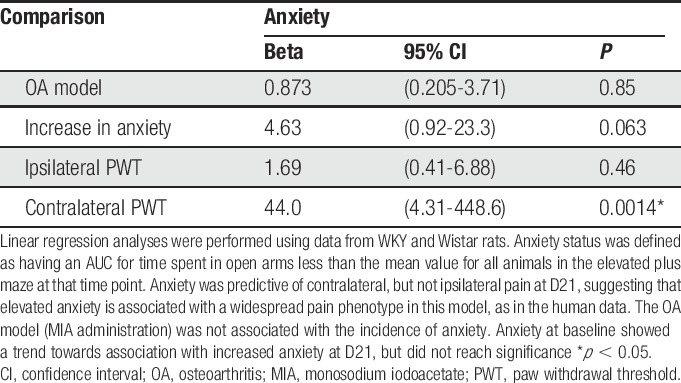
Association between anxiety, OA status, and pain behaviour in the preclinical WKY-MIA model of OA.

### 3.3. Increased spinal and supraspinal glial cell responses

**Figure 3.**
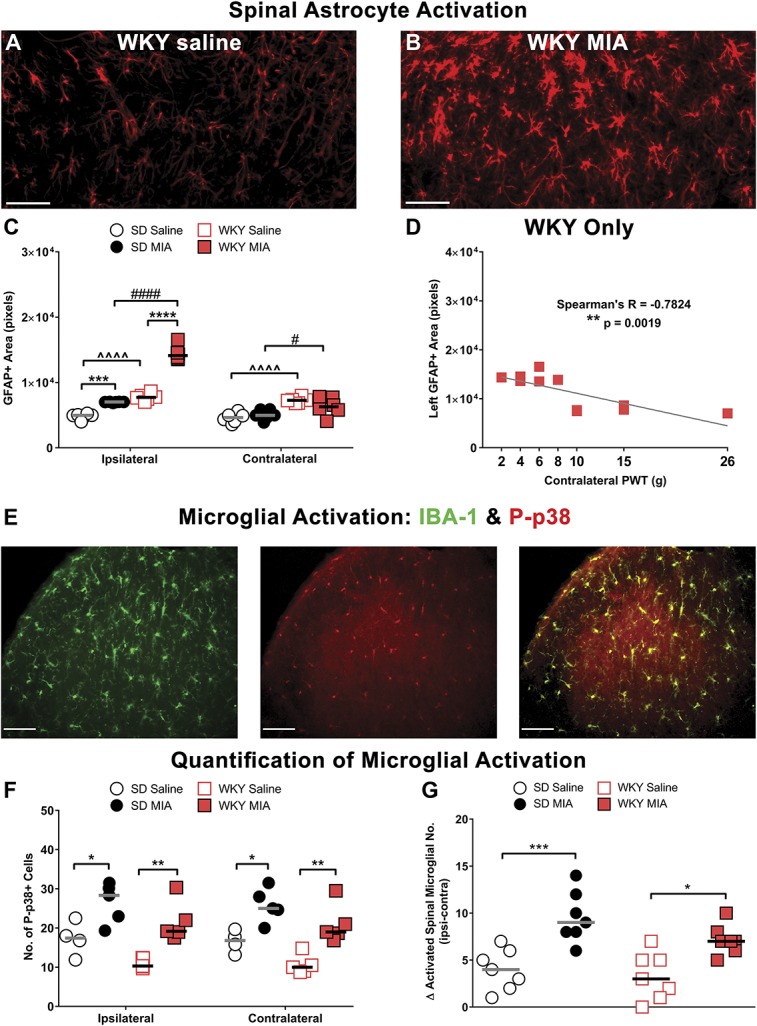
Enhanced MIA-induced pain behaviour in the WKY rat is accompanied by activation of spinal astrocytes, but not microglial cells. Representative images of astrocyte activation visualised through GFAP immunofluorescence in the ipsilateral spinal cord dorsal horn in the WKY strain 21 days after intra-articular injection of saline (A) or MIA (B). Quantification revealed MIA-induced astrocyte activation in the ipsilateral spinal cord of both SD and WKY rats when compared with saline-treated controls (C), but changes in GFAP expression were more pronounced in WKY rats, and spinal GFAP expression levels were significantly higher bilaterally in both saline and MIA-treated WKY rats compared with their SD counterparts. GFAP expression (defined as GFAP+ area) was significantly correlated with decreased contralateral PWTs in the WKY strain (D), suggesting a clear association between astrocyte activation and augmented pain behaviour. GFAP+ area was calculated as the number of pixels with intensity >55 in each area. Scale bars = 50 µm. Data are mean ± SEM. ***P < 0.001, ****P < 0.0001 vs saline control, #*P* < 0.05, ####*P* < 0.0001 vs SD-MIA, ^^^^*P* < 0.0001 WKY-MIA vs SD-MIA. By contrast, no strain differences were observed in spinal microglial activation assessed through P-p38 expression (E and F) or morphological analyses (G). Representative spinal cord image demonstrating P-p38 (red) expressed almost exclusively in IBA-1–positive (green) activated microglial cells (E). Quantification revealed a similar bilateral activation in P-p38–expressing spinal microglial cells after MIA injection in both SD and WKY rat strains (F). Morphological analysis also revealed a similar MIA-induced increase in ipsilateral microglial activation in both strains (G). Data are mean ± SEM, analysed using 1-way (microglia) or 2-way (P-p38) analysis of variance with Bonferroni post hoc testing. *P < 0.05, **P < 0.01, ***P < 0.001 vs respective saline-treated controls. (n = 5 rats per group, 6-7 sections per rat). GFAP, glial fibrillary acidic protein; MIA, monosodium iodoacetate; PWT, paw withdrawal threshold; SD, Sprague-Dawley.

**Figure 4.**
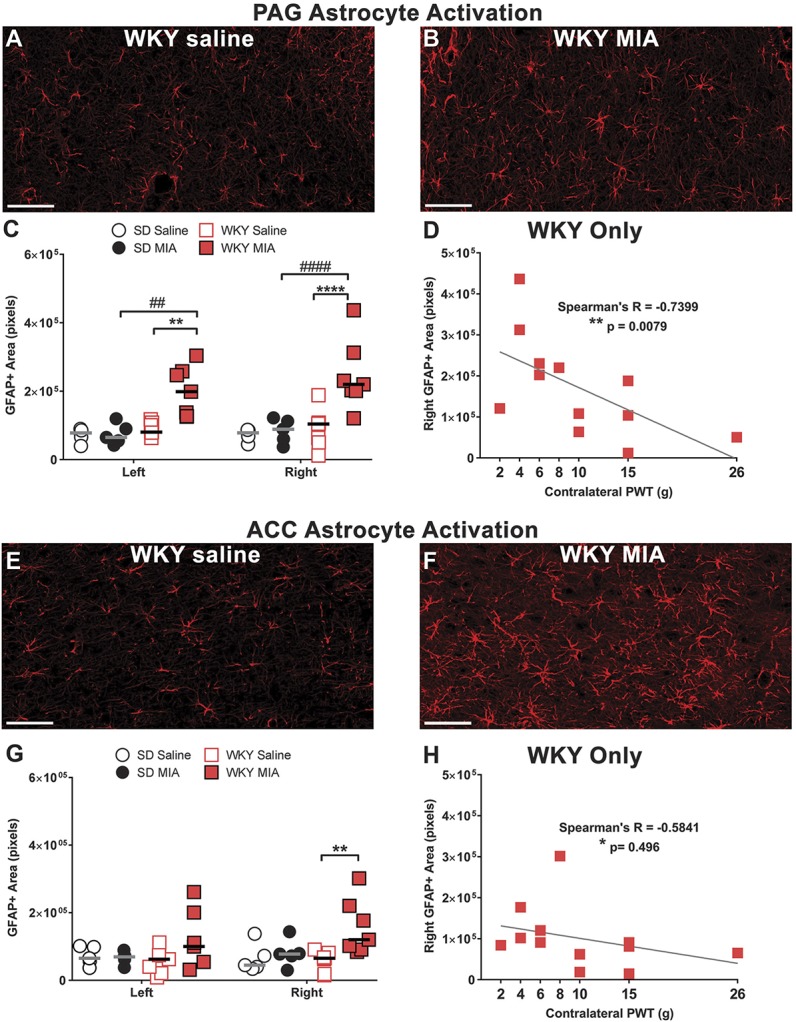
Monosodium iodoacetate–induced pain behaviour in the WKY rat is accompanied by activation of supraspinal astrocytes not seen in normo-anxiety SD rats. Representative images demonstrate increased GFAP immunofluorescence in the right PAG (B) and ACC (F) in the WKY strain compared with saline-treated controls (A and E, n = 5/group). Quantification revealed MIA-induced astrocyte activation bilaterally in the PAG (C), and unilaterally in the ACC (G), of WKY, but not SD rats (see Figure S3, available at http://links.lww.com/PAIN/A696). GFAP expression (defined as GFAP+ area) in the right PAG and right ACC correlated with contralateral PWTs in the WKY strain (D and H), suggesting a clear association between supraspinal astrocyte activation and augmented pain behaviour in the WKY strain. GFAP+ area was calculated as the number of pixels with intensity >55 in each area. Scale bars = 50 µm. Data are mean ± SEM. **P < 0.01, ****P < 0.0001 vs saline control, ##*P* < 0.01, ####*P* < 0.0001 vs SD-MIA. ACC, anterior cingulate cortex; GFAP, glial fibrillary acidic protein; MIA, monosodium iodoacetate; PAG, periaqueductal gray; PWT, paw withdrawal threshold; SD, Sprague-Dawley.

Given the well-established contribution of neuroimmune cells to central sensitization and the transition to chronic pain, we probed the role of glial cell activation in pain-associated CNS regions. Astrocyte and microglial markers were assessed in the dorsal horn of the spinal cord (Fig. 3) and brains (Fig. 4) from WKY and SD rats. A low-magnification image of GFAP labelling in the spinal cord dorsal horn and regions used for quantification can be found in the supplementary material (Figures S2 & S3, available at http://links.lww.com/PAIN/A696). Quantification of GFAP immunofluorescence, a marker of astrocytes, revealed higher levels bilaterally in the dorsal horn of the spinal cord in saline-treated WKY rats (WKY-sal), compared with their SD counterparts (SD-sal, Figs. 3A and C). Importantly, ipsilateral dorsal horn GFAP immunofluorescence was increased to a significantly greater extent in WKY-MIA rats at 28 days (Figs. 3B and C). No changes in GFAP were observed in the contralateral dorsal horn of WKY-MIA rats, compared to WKY-sal (Fig. 3C). However, lowered contralateral PWTs were correlated with GFAP immunofluorescence in the ipsilateral dorsal horn in WKY-MIA rats (Spearman's *R* = −0.78, *P* = 0.0019, Fig. 3D).

By contrast, we observed no significant differences in MIA-induced microglial activation between the 2 strains (Figs. 3E–G). An increase in microglial activation was observed in the ipsilateral spinal cord of both strains (Fig. 3G and S4, available at http://links.lww.com/PAIN/A696), with a bilateral increase in P-p38+ microglial cells (Figs. 3E and F), but no significant difference between strains. These data replicate our previous work demonstrating MIA-induced activation of microglial cells in the spinal cord,^42^ but suggest that elevated anxiety does not lead to increased microglial activation in the spinal cord.

Because anxiety is mediated supraspinally, we also investigated changes in astrocyte activation in pain-associated regions of the brain. We observed a bilateral increase in GFAP immunofluorescence in the ventrolateral PAG (Figs. 4A–C) and ACC (Figs. 4E–G) in WKY-MIA rats, but not in SD-MIA rats (Figure S3, available at http://links.lww.com/PAIN/A696; & Figs. 4C and G). Lowered contralateral PWTs in MIA-treated WKY rats were correlated with GFAP immunofluorescence in both the ventrolateral PAG (Spearman's *r* = −0.74, *P* = 0.0079, Fig. 4D) and ACC (Spearman's *r* = −0.58, *P* = 0.0496, Fig. 4H).

To consolidate the link between altered PWTs and increased GFAP immunofluorescence, an intervention study was performed. Duloxetine (30 mg/kg, subcutaneous) reversed established reductions in ipsilateral and contralateral PWTs in WKY-MIA rats (Table 4). This effect was associated with significantly lower GFAP immunofluorescence in the dorsal horn of the spinal cord and ventrolateral PAG of WKY-MIA rats. There was no change in the level of GFAP immunofluorescence in the ACC (Table 4). In SD-MIA rats, duloxetine reversed weight-bearing asymmetry, but had no effect on the contralateral PWTs (data not shown). Our data demonstrate increased basal, and MIA-induced, astrocyte activation associated with an altered pain phenotype in the WKY strain, and that both can be reversed by a centrally acting anxiolytic.

**Table 4:**
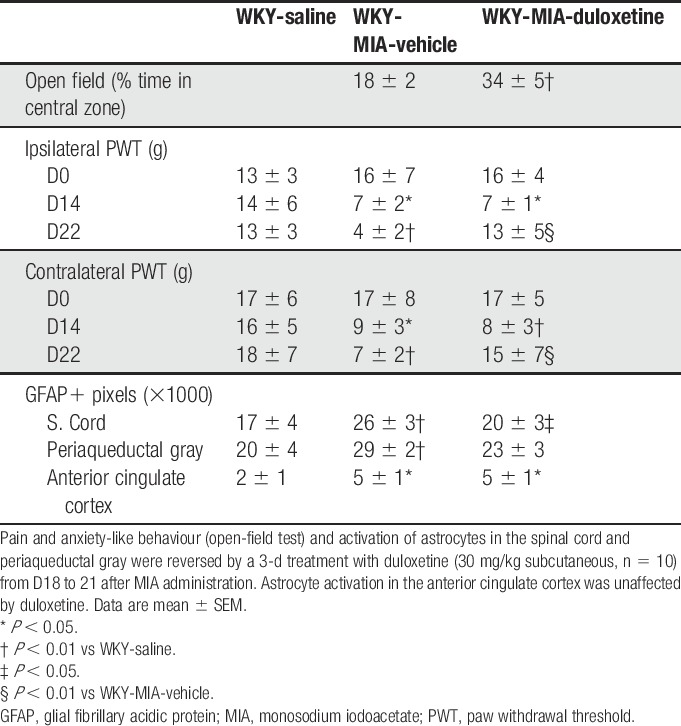
Duloxetine reverses anxiety-like behaviour and reduces MIA-induced pain behaviour and astrocyte activation in a rat model of high anxiety.

## 4. Discussion

In this study, we show that higher anxiety scores, but not depression scores, are significantly associated with higher pain sensitivity in individuals with knee OA. In addition, after adjusting for the effect of depression, anxiety score at baseline was associated with increased risk of knee pain at 12 months. Anxiety at baseline was also associated with augmented pain behaviour in a rodent model of OA. In this model, we demonstrated increased activation of a marker of astrocytes in the PAG and ACC in the high-anxiety OA model. Pharmacological attenuation of existing OA behavioural pain responses in the high-anxiety model was associated with a decrease in the glial cell response in the PAG, suggesting a role for astrocytes in the link between anxiety and OA pain.

### 4.1. Osteoarthritis and anxiety associated with augmented pain responses

The lowered PPTs at both the OA joint (knee) and remote sites (sternum) in our study suggest altered central pain processing in individuals with OA, consistent with previous reports.^2,37^ Herein, we demonstrate that individuals with OA and high anxiety reported greater pain and had lowered PPTs as assessed by QST, compared to individuals with OA and normal anxiety levels. Associations between anxiety and PPTs, and self-reported pain in the knee OA cohort remained significant after adjustment for depression. Our novel findings are consistent with previous data showing correlated self-reported pain and anxiety scores and increased disability in an outpatient population^3^ and community population^5^ with OA and anxiety or depression.

To better understand the longer-term impact of anxiety on knee pain, an analysis of a large number of individuals from the general population was undertaken. This revealed that high anxiety scores predicted the onset of knee pain over a 1-year period, but knee pain at baseline did not statistically alter the onset of high anxiety. A recent study reported that moderate to very severe pain was associated with a higher risk of mood or anxiety disorders at 3 years,^13^ indicating the importance of early and successful treatment interventions for people with OA knee pain. Taken together, our findings support the hypothesis that anxiety drives an increase in sensory pain thresholds both at the site of disease and remote sites, and an individual's self-reporting of pain. Our findings have wide-ranging implications for the treatment of comorbid pain and anxiety, and may account for why individuals with high anxiety and OA have a reported higher level of opioid use.^5,38^

### 4.2. Anxiety is associated with spread of pain to remote sites in the WKY-MIA model of anxiety and osteoarthritis pain

Before induction of the model of OA, WKY rats exhibited a heightened behavioural anxiety–like profile, as expected.^18,34^ In our study, behavioural scores of anxiety at baseline were a predictor of pain behaviour after induction of the MIA model of OA, validating the utility of the WKY strain of rat to investigate the mechanisms mediating the influence of anxiety on OA pain. The measure of mechanical hypersensitivity (reduced ipsilateral PWTs^43^) was greater in WKY-MIA than in SD-MIA. WKY-MIA rats also had lowered PWTs in the contralateral hind paw, replicating the remote pain reported clinically in OA^2^ and in our clinical study of individuals with OA and high anxiety levels. By contrast, the surrogate behavioural test of pain on loading (weight-bearing) was comparable between the 2 strains in the model of OA pain. This likely reflects the influence of bilateral lowering of PWTs in the WKY rats, which confounds tests dependent on a shift in weight bearing from the injured to the uninjured side.

Given the established role of astrocytes in preclinical models of musculoskeletal pain,^42,49^ and in the transition from acute to chronic pain states,^18,24^ the potential role of these glial cells was investigated. In the model of OA pain, GFAP immunofluorescence was significantly increased in the ipsilateral spinal dorsal horn in both strains of rats, but to a significantly greater extent in the WKY rats. Although spinal cord microglia have an established role in sensitization of pain processing^36^ and are activated in this model of OA pain,^42^ we found no differences in the numbers of activated microglia in the ipsilateral spinal cord between the SD-MIA and WKY-MIA rats. These data suggest that, at this time point at least, astrocytes in the spinal cord play a more prominent role in heightened OA pain responses associated with anxiety.

Within the brain, there was a significant bilateral increase in GFAP immunofluorescence in the ventrolateral PAG and a unilateral increase in the right ACC in WKY-MIA rats, but not in SD-MIA rats. The ACC has been identified as a key structure in anxiety–chronic pain interactions, mediating both pain-driven increases in anxiety and subsequently enhanced pain, through distinct mechanisms.^61^ As glial activation is associated with increased neural activity, and differences in astrocytes in specific brain regions were correlated with the pain behaviour specific to WKY-MIA rats (contralateral lowering of PWTs), these data suggest astrocytes in the ACC and PAG are potential drivers of the CNS mechanisms by which anxiety contributes to pain phenotype in this OA model. Indeed, recent studies have pointed to a key role for astrocyte activation in the generation of contralateral pain phenotypes in mouse models of neuropathic pain,^22,28^ unmasked by a shift in the balance between excitation and inhibition. A mechanism such as this may be of particular relevance to the spread of OA pain to remote sites, which is likely a centrally mediated phenomenon.

To better understand the potential associations and mechanisms, we undertook a pharmacological study with duloxetine, which acts centrally to increase synaptic levels of serotonin (5-HT) and noradrenaline (NA) in numerous brain regions^15,26^ and has analgesic effects in chronic pain states.^31,40^ Duloxetine reversed both the contralateral pain phenotype specific to WKY-MIA rats and the increased bilateral GFAP immunofluorescence in the ventrolateral PAG, but had no effect on GFAP in the right ACC of WKY-MIA rats. These data suggest that the effects of duloxetine on anxiety-driven OA pain are occurring at the level of the brainstem, which would be consistent with the known role of this region in driving the descending inhibitory control pathways.^39^ Although numerous studies have demonstrated short-term effects of duloxetine on astrocytes in the spinal cord in models of pain,^10,30,44^ our study is the first to report of an effect of duloxetine on astrocytes in a pain-associated brain region. Our findings support a role of central mechanisms driving facilitated OA pain responses under conditions of high anxiety, and consolidate the link between astrocyte activation in discrete brain regions and contralateral pain behaviour.

### 4.3. Study limitations

We note several limitations with this study that are worth considering when evaluating the results. In our clinical data, we did not assess the role of anxiety on pain thresholds in pain-free individuals with radiographic OA; hence, we cannot draw conclusions regarding nonpainful radiographic OA. However, although the effects of anxiety on PPTs were not significant in control individuals, this could be due to the smaller number of high-anxiety, knee pain-free participants included in this study. For some sites, effects of anxiety on PPTs were considerably larger in knee OA participants than in controls (eg, medial PPTs knee OA participants = 0.59 StDev, control participants = 0.37 StDev). However, anxiety-induced differences in sternum PPTs were virtually identical in knee OA cases (0.48 StDev), and controls (0.47 StDev), and the lack of statistical significance in the latter group here can be accounted for by a lack of power. Finally, we assessed all PPTs over bone, and findings may not be generalizable to the wider musculoskeletal field where PPTs are sometimes assessed over muscle.^17^

There are also some limitations to the preclinical work. Male rats only were used for this study to reduce intragroup variability while developing the model and to maintain consistency with our previous work.^43^ However, this reduces the translational value of the data because ∼60% of our clinical subjects were female, OA is more prevalent in the female population, and females tend to have more severe knee OA.^48^ It will be important to determine whether there are any sex-specific effects in this model of anxiety and OA-like pain in future studies.

## 5. Conclusion

Pain pressure thresholds and anxiety scores in people with knee OA are highly associated, and anxiety at baseline predicts future knee OA 1 year later. Our clinical data support the investigation of new targets for treating pain in high-anxiety patients with OA. We have successfully developed a rodent model of comorbid anxiety and OA, in which anxiety at baseline was associated with greater pain behaviour and an increase in activation of astrocytes in the PAG and ACC brain regions in the model of OA. The sensitivity of both pain responses and glial cell activation in the PAG to pharmacological intervention in this model suggests a potential role of the astrocytes in the link between anxiety and OA pain.

## Conflict of interest statement

This work was supported by Arthritis Research United Kingdom (grant numbers 18769, 20777). All authors state no other conflict of interest.

## Appendix A. Supplemental digital content

Supplemental digital content associated with this article can be found online at http://links.lww.com/PAIN/A696.
